# Physical Activity Among Medical Students at the University of Khartoum, Sudan, 2022: Knowledge, Practice, and Barriers

**DOI:** 10.7759/cureus.45914

**Published:** 2023-09-25

**Authors:** Mohamed H Fadul, Abdalla Fadul, Abdullatif Yasir H Eissa, Ahmed Zaki W Mohamed Elhassan, Gaffar Alemam A Manhal, Rania H Abdelgafour

**Affiliations:** 1 Medical School, Faculty of Medicine, University of Khartoum, Khartoum, SDN; 2 Internal Medicine, Hamad Medical Corporation, Doha, QAT; 3 Community Medicine, Faculty of Medicine, University of Khartoum, Khartoum, SDN

**Keywords:** exercise, physical inactivity, medical students, lifestyle, sedentary behaviour, physical activity, sudan

## Abstract

Introduction

Physical inactivity is a highly prevalent modifiable risk factor for many diseases, including cardiovascular and cerebrovascular diseases, the leading causes of death. Many health institutes have issued and adopted guidelines and recommendations on physical activity. This study aims to study the knowledge, practice, and barriers of medical students enrolled at the University of Khartoum, Sudan, regarding physical activity. It aimed to assess the students’ knowledge about physical activity, to determine the levels of physical activity and compare it with the WHO recommended levels and data from other countries, to compare the activity and sedentary levels between males and females, and to take a look on the barriers to physical activity.

Methods

An observational, descriptive, cross-sectional study was conducted at the Faculty of Medicine, University of Khartoum in December 2022. A total of 336 students were included using systematic random sampling. Data were collected using an online questionnaire that included the Global Physical Activity Questionnaire (GPAQ). Levels of physical activity were described and compared between males and females.

Results

Around 40.5% of the students achieved the recommended level of physical activity. The mean level of vigorous-intensity physical activity was 50.9 minutes/week (SD= 125.7), the mean level of moderate-intensity physical activity was 156.5 minutes/week (SD= 316.1), the mean level of total physical activity was 207 minutes/week (SD= 356). Between males and females, there was a significant mean difference in the level of vigorous-intensity physical activity and total physical activity. The mean level of sedentary behavior was 7.61 hours/day (SD= 4.62) with no significant difference between males and females (p=0.127). Students showed good knowledge about the cardiovascular and mental benefits of physical activity but not about its protective benefits against cancer. Only 19.4% knew the WHO-recommended levels of physical activity. The most common major barriers toward physical activity reported by the students were lack of time (43.8%), and lack of suitable facilities (31.3%) among others.

Conclusion

More than half (59.5%) of the students were insufficiently active. Levels of physical activity were significantly associated with the participants’ sex but not their knowledge of the recommended levels of physical activity. Males showed higher levels of physical activity. Lack of time was the most common barrier to exercise. More knowledge and education about physical activity should be provided as well as suitable facilities.

## Introduction

In the modern era, the lifestyle of most people around the world has changed. Inactive sedentary behavior has become more common among different age groups at home, work, and during leisure time. Also, passive means of transportation increase physical inactivity. The World Health Organization (WHO) reports that more than 25% of the world’s adult population is insufficiently active. The percentage is much higher in adolescents; around 81% of this population do not meet the WHO recommendations for aerobic activity [1]. Notably, because urbanization and industrial development allow for more physical inactivity, levels of inactivity are higher in high-income countries [2,3]. However, this increase in inactive lifestyles has been accompanied by a significant change in the pattern of diseases that cause immense morbidity and mortality, as physical inactivity is a modifiable risk factor for cardiovascular and cerebrovascular diseases, the two most common causes of death in the Western world [4]. A report suggested that physical inactivity is directly responsible for 5.3 million deaths per year [5]. Indeed, some reviews report that physical inactivity has the highest prevalence among all other modifiable risk factors of cardiovascular disease [4]. Due to its modifiable nature and high prevalence, physical inactivity warrants more research and intervention efforts.

The WHO defines physical activity as “any bodily movement produced by skeletal muscles that requires energy expenditure” [1]. It has many varieties, including aerobic or endurance activities, muscle-strengthening activities (such as weight lifting and resistance exercises), and bone-strengthening (weight-bearing) activities. Regular physical activity improves different aspects of health including brain health and cognition, musculoskeletal health, and immunity. Many other components (e.g. self-esteem, body image, and quality of life) are also improved with regular physical activity [6, 7]. The association between physical activity and decreased levels of obesity and type 2 diabetes is well established. Furthermore, it has been proven that physical activity reduces the risks of cardiovascular disease, stroke, anxiety, depression, dementia, and many cancers [8, 9]. Physical exercise has been deemed 'the miracle drug' owing to its numerous established advantages and the potential for many more that remain unproven [10]. The evidence supporting this claim is overwhelming and still growing. Unfortunately, despite these benefits, levels of physical activity all over the world remain inadequate. This encouraged the WHO to develop the "Global Action Plan on Physical Activity 2018-2030", with a goal of a 10% reduction in physical inactivity levels by the year 2025, and 15% by the year 2030 [11].

Sedentary behavior as defined by the U.S. Department of Health and Human Services (HHS) refers to “any waking behavior characterized by a low level of energy expenditure while sitting, reclining, or lying” [7]. It has been linked to an increased incidence of cardiovascular disease and cancer [3]. One of the major contributors to sedentary behavior is the time spent in front of screens. Literature shows evidence of a consistent adverse association between sedentary screen time and mental health [12].

In Sudan, a low-income country, levels of inactivity and sedentary behavior may be less than in higher-income countries. Nevertheless, sports and regular exercise are not that common in Sudanese communities. Most of the physical activity may be from work, public transportation, or other daily activities. However, this may be insufficient and levels of physical activity may still fall short of the recommended guidelines [13, 14].

Many researchers around the world study the levels of physical activity and levels of sedentary behavior in the general population and different population groups. Specific groups, such as university students, medical students, and healthcare professionals, get more attention. Many studies focused on university students since they are prone to high levels of physical inactivity and because of the criticality of this stage of life where habits and long-term lifestyles can be built [15]. Moreover, some studies demonstrated that in the first years of university, students gain additional weight. This was called by some authors as the "freshman weight-gain phenomenon" [16-18]. For example, studies from the UK [19], Peru [20], Saudi Arabia [21], Iran [22], Thailand [23] and other countries have investigated the levels of physical activity in university students. Many of these studies concluded that levels of physical activity in these university students are insufficient. In Sudan, Yousif et al. used the International Physical Activity Questionnaire (IPAQ) and found low levels of physical activity among medical students at Al-Neelain University [24]. In many studies, the major barriers to regular physical activity were lack of time, the unavailability of suitable places and facilities, health issues such as musculoskeletal injuries, and cardiorespiratory deficits among other causes [19, 21, 25].

According to the 2020 WHO Guidelines on Physical Activity and Sedentary Behavior, adults aged 18-64 should do at least 150-300 minutes of moderate-intensity aerobic physical activity, or at least 75-150 minutes of vigorous-intensity aerobic physical activity, or an equivalent combination of the two throughout the week for substantial health benefits. For additional health benefits, adults should also do muscle-strengthening activities involving all major muscle groups at least two days a week. Furthermore, adults are advised to do more than these recommended levels. They also recommend decreasing the amount of inactivity time that is spent in sedentary behavior [3].

This study provides a descriptive view of the levels of physical activity and sedentary behavior among medical students at the University of Khartoum, evaluates them against the WHO-recommended levels, compares them between different student groups, and collates them with data from other universities.

## Materials and methods

A descriptive cross-sectional study was conducted at the Faculty of Medicine, University of Khartoum, Sudan in December 2022. The study population was students at the faculty aged 18 years or older, as the adopted WHO guidelines are for adults aged 18-64.

The sample size was calculated using Epi Info statistical software (Centers for Disease Control and Prevention, Atlanta, GA, USA). Population (N) was estimated as 2500 (number of faculty students), the confidence interval (CI) was set to 95%, and the margin of error to 5%. The calculated sample size (n) was 334 participants. A list of faculty students was used as a sampling frame from which participants were randomly selected via systematic random sampling and an online random number generator, with a sampling interval of 7. This sampling interval was calculated by dividing the total number of students by the desired sample size (N/n).

Data were collected using an online Google Forms questionnaire constructed by the authors. Selected participants were sent the questionnaire link via text message. A pilot study of medical students (n=7) was conducted to assess and evaluate the online questionnaire for technical or any other issues. The questionnaire contained a section on participants' sociodemographic data (age, sex, and year of study), and their knowledge and perceived barriers toward physical activity. Section 2 comprised the validated "Global Physical Activity Questionnaire (GPAQ)" which was developed by the WHO as a part of the WHO's stepwise approach to non-communicable diseases risk factor surveillance. The GPAQ consists of 16 items to determine the amount of time spent doing vigorous or moderate-intensity physical activity at work (six items), transportation (three items), and leisure time (six items), in addition to the amount of time spent in sedentary behavior (one item) [26].

Data were cleaned, managed, and analyzed using Microsoft Office Excel 2016 (Microsoft Corp., Redmond, WA, USA) and the Statistical Package for Social Sciences (SPSS) version 28 (IBM Corp., Armonk, NY, USA). The characteristics of the study population were described in means, standard deviations, frequencies, and percentages. Knowledge about, and barriers to, physical activity were described in percentages and frequencies, and presented in graphs.

Levels of physical activity were measured in minutes per week, and expressed in means and standard deviations. Levels of total physical activity were calculated from levels of vigorous- and moderate-intensity activities using metabolic equivalents (MET). Then, students were categorized as either achieving the WHO-recommended levels or not, and the percentages were illustrated in graphs. Levels of sedentary behavior were measured in hours per day, described by means and standard deviations, and expressed in a histogram. 

T-test was used to compare mean levels of physical activity and sedentary behavior between males and females. The chi-square test and odds ratio were used to assess the relationship of levels of physical activity with two independent variables: sex, and knowledge about the WHO-recommended levels of physical activity.

Ethical approval was attained from the Ethical Committee of the Department of Community Medicine, Faculty of Medicine, University of Khartoum. Informed consent was taken from participants before they filled out the questionnaire. Those who refused to participate were excluded.

## Results

A total of 336 medical students participated in the study. The mean age of the participants was 20.7 years (SD= 1.95). Around 19% of the participants were in the second year (third semester), 17.3% were in their first year, 16.1% were in the second year (fourth semester), 15.2% were in the fourth year, 14% were in the fifth year, 11% were in the third year (sixth semester) and 7.4% were in their final sixth year. One-third (33.3%, n=112) of the participants were males and 66.7% (n=224) were females.

**Figure 1:**
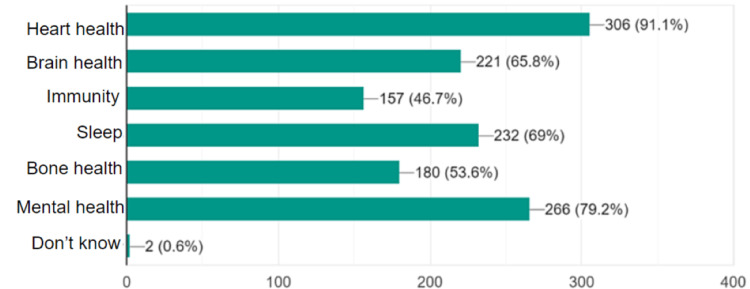
Knowledge about health aspects that can be improved by physical activity among medical students at the University of Khartoum, 2022 (n=336).

The participants were asked: "According to your knowledge, physical activity can improve which of the following?" Around 91% of the respondents were aware that physical activity could improve heart health. A majority were also aware that physical activity positively impacted mental health (79%), sleep quality (69%), brain health (69%), and bone health (54%). However, only 47% were aware that physical exercise improved immunity against different infections. Around 0.6% chose “don’t know” (Figure 1).

**Figure 2:**
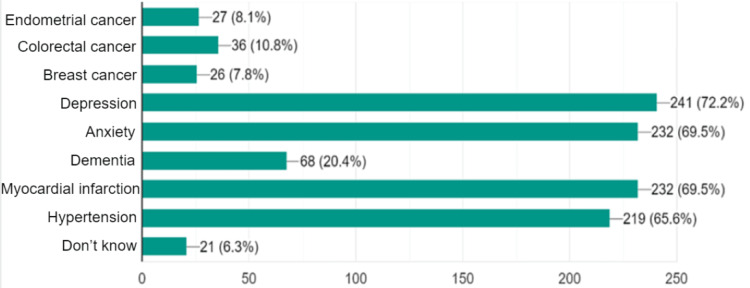
Knowledge about health conditions that can be prevented by regular physical activity among medical students at the University of Khartoum, 2022 (n=336).

The participants were asked: "According to your knowledge, physical activity can decrease the risk of which of the following?" A majority (72%) knew that physical activity could prevent depression, anxiety (70%), myocardial infarction (70%), and hypertension (66%). However, a minority were aware of links between physical activity and dementia (20%), colorectal cancer (11%), endometrial cancers (8%), and breast cancer (8%). Around 6.3% chose “don’t know" (Figure 2).

The mean level of moderate-intensity physical activity was 156.5 minutes/week (SD= 316.1), with no significant difference between males and females, t(197)=9.5, p= 0.34. The mean level of vigorous-intensity physical activity was 50.9 minutes/week (SD= 125.7). It was 19.5 minutes/week (SD= 69.3) for females and 113.6 minutes/week (SD= 179.1) for males. Independent sample t-test showed a significant mean difference of 94 minutes/week (95% CI: 121-67), t(334)=6.9, p<0.001 (Table 1).

**Table 1:** Independent t-test showing significant difference in mean levels of vigorous-intensity physical activity between males and females among medical students at the University of Khartoum, 2022 (n=336)

	Sex	N (%)	Mean	Std. Deviation	Std. Error Mean	t (df)	P value
Levels of vigorous physical activity (min/week)	Male	112 (33)	113.6	179.1	16.9	6.9 (334)	<0.001
	Female	224 (66)	19.5	69.3	4.6		

The mean level of total physical activity was 207 minutes/week (SD=356). A two-sample t-test was performed to compare the mean levels of physical activity between females (mean, 164 minutes/week, SD= 315) and males (mean, 294 minutes/week; SD= 414). There was a significant difference of 130.3 (95% CI; 210-50) in the mean level of total physical activity between males and females, t(334)= 3.2, p= 0.001 (Table 2).

**Table 2:** Two samples t-test showing the difference in mean levels of total physical activity between males and females among medical students at the University of Khartoum, 2022 (n=336).

	Sex	N (%)	Mean	Std. Deviation	Std. Error Mean	t (df)	P value
Levels of total physical activity (min/week)	Male	112 (33)	294.3	414.1	39.1	3.2 (334)	0.001
	Female	224 (66)	163.9	315.0	21.0		

**Figure 3:**
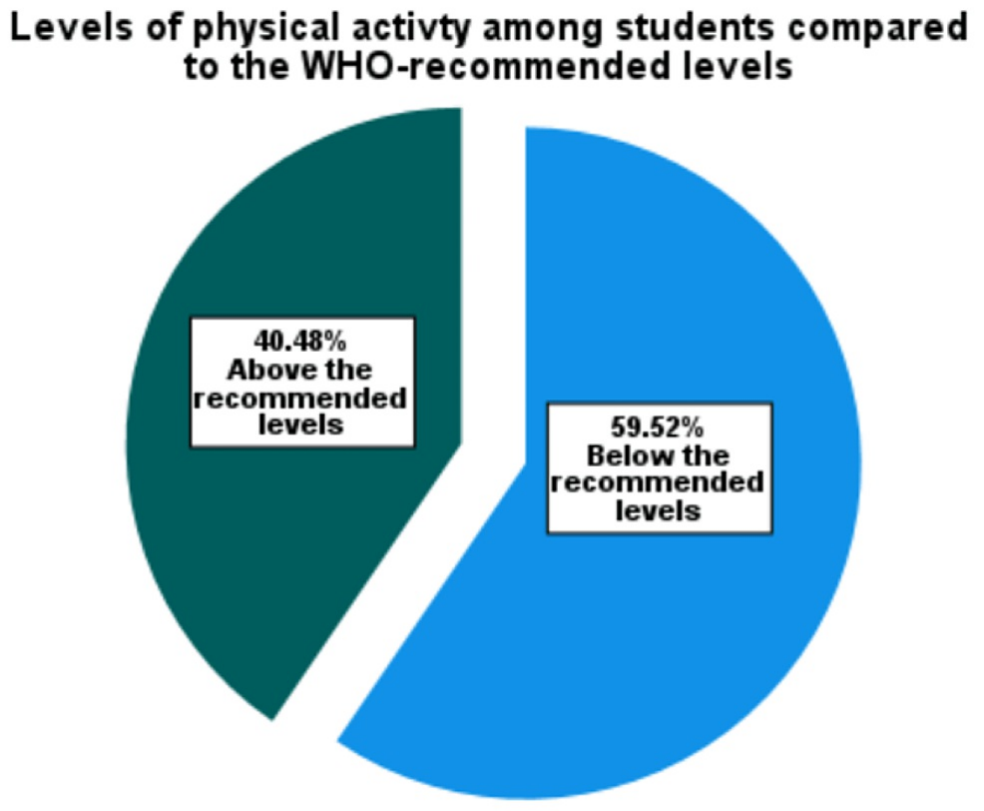
Percentage of medical students achieving the WHO-recommended levels of physical activity or more (labeled as "above") at the University of Khartoum, 2022 (n=336)

The measured levels of total physical activity among students were compared against the WHO-recommended levels, and the participants were then categorized as either achieving the recommended levels or not. Around 40.5% (n=136) of the students achieved the recommended levels of physical activity, and 59.5% (n=200) did not (Figure 3).

**Figure 4:**
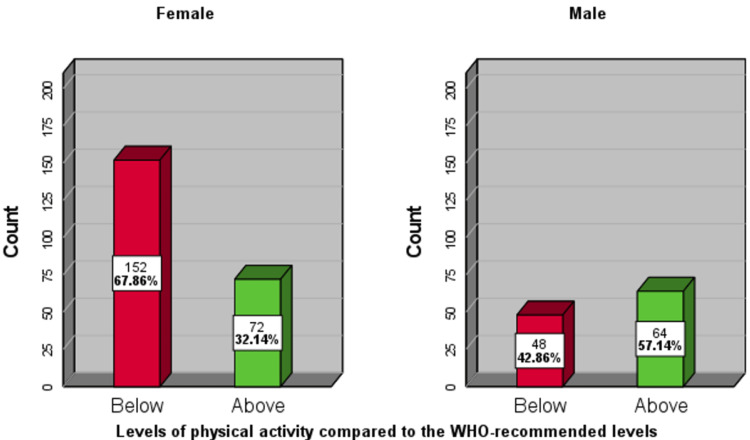
Percentages of medical students achieving the recommended physical activity levels or more (labeled as 'above') at the University of Khartoum, 2022 (n=336). Left panel: females; right panel: males

Among the male students, 57% (n=64) achieved the WHO-recommended levels of physical activity, while 43% (n=48) did not. Among the female students, 32% (n= 72) achieved the WHO-recommended levels, while 68% (n= 152) did not (Fig 4).

Chi-square tests showed a significant relationship between sex and achievement of the recommended level of physical activity ( X^2^(1)=19.4, p<0.001), odds ratio= 2.8 (95% CI= 1.8-4.5); But no significant association between levels of total physical activity and knowledge about the WHO-recommended levels (X^2^(1)=0.57, P=0.449) (Table 3).

**Table 3:** Relationship of levels of physical activity with two independent variables (sex and students' knowledge about the WHO-recommended levels of physical activity), 2022 (n=336).

		Levels of physical activity in relation to the WHO-recommended levels		
		Below N (%)	Above N (%)	P value
Sex	Female	152 (76)	72 (53)	<0.001
	Male	48 (24)	64 (47)	
	Total	200 (100)	136 (100)	
Knowledge of recommended levels	Incorrect	164 (82)	107 (79)	0.449
	Correct	36 (18)	29 (21)	
	Total	200 (100)	136 (100)	

**Figure 5:**
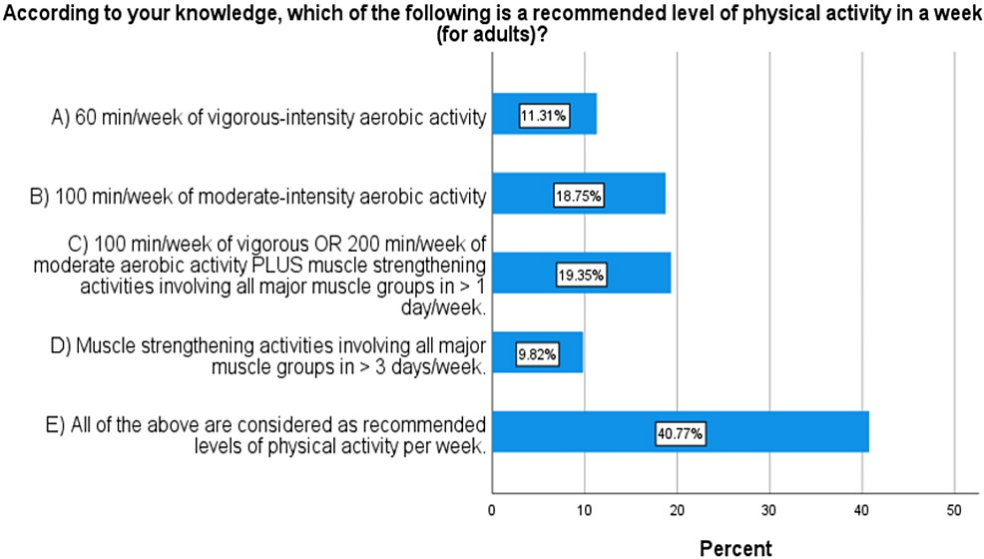
Knowledge about the WHO-recommended levels of physical activity among medical students at UofK, 2022, (n=336). The correct answer is C.

The participants were asked: "According to your knowledge, which of the following achieves the recommended levels of physical activity in a week?" Around 19.3% (n=65) chose the correct answer, and 80.7% (n=271) chose other incorrect answers (Figure 5).

**Figure 6:**
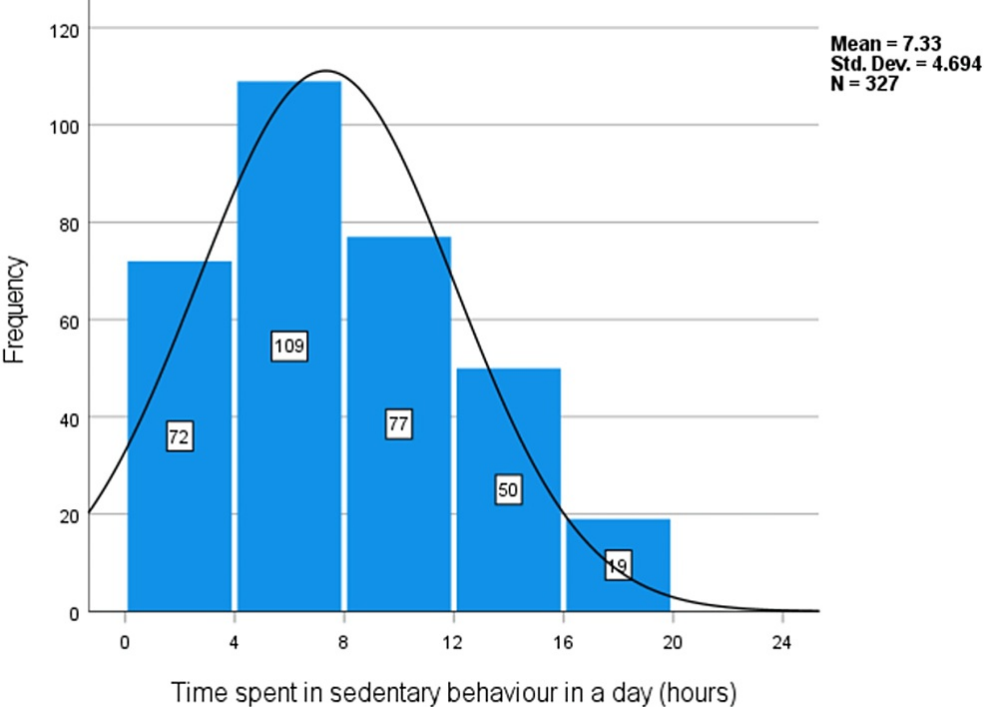
Levels of sedentary behavior (hours/day) among medical students at the University of Khartoum, 2022 (n=327, missing=9, total=336).

The mean level of sedentary behavior was 7.33 hours/day, SD=4.7 (Figure 6). No significant difference in the mean level of sedentary behavior between males (M=6.76 hours/day, SD= 4.81) and females (M=7.61 hours/day, SD=4.62), t(211)= 1.53, p= 0.127.

**Figure 7:**
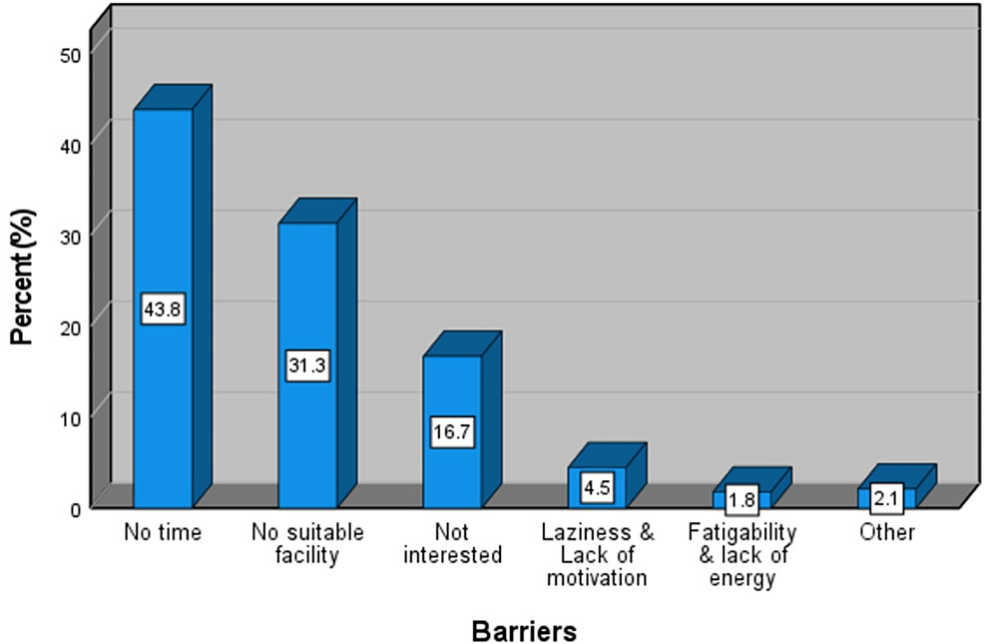
The major barriers toward physical activity perceived by medical students at the University of Khartoum, 2022 (n=336).

The major barriers toward physical activity were: lack of time (43.8%), lack of suitable facilities (31.3%), no interest in physical activity (16.7%), laziness, and lack of enthusiasm and motivation (4.5%), fatigability and lack of energy (1.8%) among others (2.1%) such as health issues and injuries (Figure 7).

## Discussion

This study investigated the knowledge, practice, and barriers of medical students at the University of Khartoum, Sudan, toward physical activity in December 2022. It aimed to assess the students’ knowledge about physical activity, to determine the levels of physical activity and compare it with the WHO-recommended levels and data from other countries, to compare the activity and sedentary levels between males and females, and to take a look at the barriers to physical activity.

The mean age of the participants was 20.7 years (SD= 2.0). Only 1.2% of the students were above 24 years and the maximum age was 26 years. The majority (66.6%) of the participants were of the female sex; this percentage is also applicable at the level of the faculty population, with other departments at the faculty reporting similar percentages.

Most of the respondents were from the second year (third semester). The sixth-year students showed the lowest response rate; only 24 sixth-year students participated in this study. This may be because of their high academic study load. Students in the third year were the second minority, this may be due to their upcoming end-of-semester examinations. These findings also emphasize that lack of time is a real major and common perceived barrier toward regular physical activity, i.e., medical students do not think they have enough time to participate in the study and fill out the online questionnaire.

Only a minority (9.0%) of the students did not know that physical activity can improve heart health (the most apparent relationship); the least-known benefit was that exercise can improve the immune system (46.7%). Regarding health conditions that can be prevented by regular physical activity, students showed a good knowledge of cardiovascular diseases (hypertension and myocardial infarction). However, the majority of students did not know that physical activity can prevent some malignancies. The academic medical curricula studied by the faculty are suggested to be the main source of students’ knowledge and information about the benefits of physical activity.

The mean level of vigorous-intensity physical activity was 50.9 minutes/week (SD= 125.7). The mean level of moderate-intensity physical activity was 156.5 minutes/week (SD= 316.1). The significant difference between males and females was in the levels of vigorous-intensity type of physical activity. In another study, walking was the only type of physical activity with no significant difference between males and females. Males showed higher levels in the other types, including vigorous intensity [27].

Of the students, 40.5% were sufficiently active as recommended by the WHO. This percentage was somehow lower than those reported from other countries, for example, 60% from the UK [19] and 50% in Thailand [23]. However, it could be reasonable to expect that students in Sudan, as a low-income country, are more active [2]. Most of the people in Sudan use public transportation which necessitates some walking between the different stations and to and from places. Nevertheless, people in low-income countries may find themselves in need of additional work time to increase their income. This may also decrease the inactivity time, especially when the added labor involves vigorous- and moderate-intensity physical activities. Indeed, this type of active work may be the only available choice for people in low-income countries. On the other hand, regular physical exercise is not common in Sudanese communities. Hence, only a few adequately equipped facilities are available. This may, at least in part, explain the lower percentage of physical activity found in this study.

As in other studies from universities around the world, females were significantly (p <0.001) less physically active than males [21, 23]. Only 32% of females were sufficiently active, whereas 57% of the males achieved the recommended levels of physical activity. In Sudan, females are less likely to have additional work with a high level of physical activity, and they tend to use more passive means of transport when possible. Moreover, they face a cultural barrier and suffer from the scantiness of suitable ladies-only facilities.

Sedentary behavior levels were of a mean of 7.61 hours in a typical day (SD= 4.62). This was very close to the levels reported in other countries. For example, a study from King Saud University reported a mean level of sedentary behavior of 7.16 hours/day (SD = 11.23) [21]. However, there was no significant difference between males and females in these levels. All medical students, regardless of their sex, have to give more time to their lectures, tutorials, studies, and other study-related tasks which are largely physically inactive.

Regarding students’ knowledge of the recommended levels of physical activity, only 19.3% of the students showed good knowledge by choosing the correct answer in the single best answer question. The WHO guidelines and recommendations on physical activity were not part of the academic curricula taught by the faculty.

The major barriers toward physical activity perceived by the students were lack of time (43.8%), and lack of suitable facilities (31.3%), among others. Lack of time was also the most common cause of physical inactivity in other similar studies such as those conducted in Saudi Arabia and the United Kingdom [19, 21, 25].

One of the limitations of the study is that it did not investigate the subtypes of activities that contributed to the time of physical activity nor the facilities where those activities were practiced.

## Conclusions

Less than half (40.5%) of the students achieved the recommended levels of physical activity, with a significant difference in the mean level of physical activity between males and females. However, there was no significant association between knowledge about and achievement of the WHO-recommended level of physical activity. The mean level of sedentary behavior was 7.61 hours/day (SD= 4.62) with no significant difference between males and females. Students showed good knowledge about the cardiovascular and mental benefits of physical activity. However, a minority of them knew about the benefits of physical activity in lowering the risks of cancers. Only 19.4% correctly knew the WHO-recommended levels of physical activity. The most common barriers toward physical activity reported by the students were lack of time and lack of suitable facilities.

Knowledge about and levels of physical activity need to be raised among medical students. We recommend providing more education about physical activity including its benefits and recommended levels. Since lack of time is the most common barrier toward physical activity, the education provided must assure and emphasize that some physical activity is better than none even if below the recommended levels, and that both aerobic and muscle-strengthening activities are highly beneficial to the health. We also recommend providing suitable facilities for physical exercise near or at the faculty campus, to solve both the time and the facility barriers.
